# Using Hepatocellular Carcinoma Tumor Burden Score to Stratify Prognosis after Liver Transplantation

**DOI:** 10.3390/cancers12113372

**Published:** 2020-11-14

**Authors:** Dimitrios Moris, Brian I. Shaw, Lisa McElroy, Andrew S. Barbas

**Affiliations:** Box 3512, DUMC, Department of Surgery, Duke University Medical Center, Durham, NC 27710, USA; brian.shaw@duke.edu (B.I.S.); lisa.mcelroy@duke.edu (L.M.); andrew.barbas@duke.edu (A.S.B.)

**Keywords:** hepatocellular carcinoma, liver transplantation, tumor burden score

## Abstract

**Simple Summary:**

Liver transplantation (LT) is an important therapeutic option in selected patients with hepatocellular carcinoma (HCC). Tumor factors such as size and number of tumors define the eligibility criteria for LT through the Milan criteria. The tumor burden score (TBS) incorporates both tumor number and size into a single continuous variable and has been successfully used to differentiate prognosis among patients undergoing resection for HCC. Moreover, the TBS has been included in the complex predictive scores of outcomes after LT for HCC. This study evaluated the role of the TBS as a solitary simple score to predict outcomes after LT for HCC.

**Abstract:**

Liver transplantation (LT) remains a mainstay of treatment for hepatocellular carcinoma (HCC). Tumor factors such as size and number of tumors define eligibility for LT using the Milan criteria. The tumor burden score (TBS) incorporates both tumor number and size into a single continuous variable and has been used to differentiate prognosis among patients undergoing resection for HCC. The objective of the present study was to evaluate the ability of the TBS to predict overall and recurrence-free survival in patients undergoing LT for HCC. The Scientific Registry of Transplant Recipients (SRTR) was used to analyze all liver transplants for HCC, with initial tumor size data from 2004 to 2018. There were 12,486 patients in the study period. In the unadjusted analyses, patients with a high TBS had worse overall (*p* < 0.0001) and recurrence-free (*p* < 0.0001) survival. In the adjusted analyses, a high TBS was associated with a greater hazard ratio (HR) of death (HR = 1.21; 95%CI, [1.13–1.30]; *p* < 0.001) and recurrence (HR = 1.49; 95%CI [1.3–1.7]; *p* < 0.001). When we superimposed the TBS on the Milan criteria, we saw that a higher TBS was associated with a higher hazard of recurrence at values that were either all within (HR = 1.20; 95%CI, [1.04–1.37]; *p* = 0.011) or variably within (HR = 1.53; 95%CI, [1.16–2.01]; *p* = 0.002) the Milan criteria. In conclusion, the TBS is a promising tool in predicting outcomes in patients with HCC after LT.

## 1. Introduction

Worldwide, hepatocellular carcinoma (HCC) is one of the most frequent causes of cancer-related death, and HCC-associated mortality continues to increase [1,2,3]. These trends are attributed to the chronic effect of viral infections and other known risk factors such as alcohol, aflatoxin, and, most recently, non-alcoholic fatty liver disease [4,5]. Current curative treatments, including liver transplantation (LT), surgical resection, and percutaneous ablation, are able to achieve a long-term survival of more than 50% of patients at five years [6]; however, only a small number of patients with early-stage HCC are eligible for these therapies [7,8]. The selection of treatment modality is based on tumor size, location, extrahepatic spread, and underlying liver function. Although the Barcelona Clinic Liver Cancer (BCLC) staging system has been largely adopted as an algorithm to guide the decision-making process in the management of HCC, recent studies have questioned the prognostic stratification of this classification schema due to its restrictive nature, as well as the proposed treatment allocation of patients [9,10,11,12]. The current literature supports liver resection for early HCC compared to local ablative therapies as it demonstrates better five-year overall and recurrence-free survival rates [13]. Moreover, the literature supports major anatomic resection in patients with resectable HCC as it has similar morbidity and superior disease-free and overall survival rates compared to non-anatomic resections, especially among patients without cirrhosis [14].

Liver transplantation (LT) remains an attractive therapeutic option for selected patients with HCC. At present, the Milan criteria are the predominant method for defining the criteria for the eligibility of HCC patients for LT [15]. In this system, patients with a single tumor <5 cm or up to three tumors all <3 cm are eligible for LT. Patients are given a binary designation (inside vs. outside Milan criteria). An alternative metric termed the tumor burden score (TBS) was first proposed by Sasaki and colleagues [16]. In contrast to the Milan criteria, the TBS incorporates both tumor number and tumor size as continuous variables and has been demonstrated to differentiate prognosis among patients undergoing resection for colorectal liver metastases. Tsilimigras et al. recently showed that the TBS can predict outcomes after resection of HCC within and beyond the Milan criteria [17,18,19]. In the setting of LT, the TBS has been included in models such as the Hazard Associated with Liver Transplantation for Hepatocellular Carcinoma (HALT–HCC) to predict overall survival outcomes in combination with clinically relevant variables such as model for end-stage liver disease-sodium (MELD–Na), alpha-fetoprotein (AFP), year of transplantation, underlying cause of cirrhosis, neutrophil–lymphocyte ratio, history of locoregional therapy, and Milan criteria status [20].

To date, there is no modern LT cohort in HCC assessing the role of the pre-transplant TBS to predict post-transplant outcomes such as overall and recurrence-free survival. The main objective of the present study was to evaluate the ability of the TBS to predict outcomes in patients undergoing LT for HCC using a modern national U.S. transplant database.

## 2. Results

There were 12,578 LT recipients in the study period. Patients with a high TBS (≥3.1) were less likely to be female (19% vs. 24%, *p* < 0.001), less likely to be white (79% vs. 81%, *p* = 0.018), and had slightly longer cold ischemia times (median 6.0 h interquartile range (IQR) [4.8–8.0] vs. 6.0 h [4.6–7.7]; *p* = 0.017) (Table 1).

**Figure 1:**
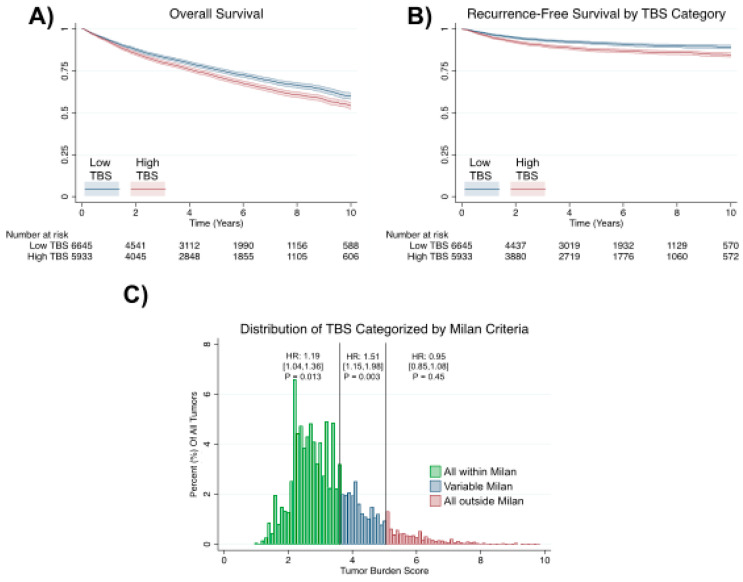
TBS and post-transplant outcomes. Patients with a high TBS had worse (**A**) overall survival, and (**B**) recurrence-free survival. (**C**) A higher TBS was associated with a higher hazard of recurrence at values that were either all within or variably within the Milan criteria. HR, hazard ratio.

All low-TBS patients were within the Milan criteria, while 26% of high-TBS patients were outside (*p* < 0.001). When plotting Kaplan–Meier curves, patients with a high TBS had worse overall and recurrence-free survival (*p* < 0.0001 for both using the logrank test; Figure 1A,B). In the Cox proportional hazard analyses, a high TBS was associated with a greater hazard ratio (HR) of death (HR = 1.21; 95%CI, [1.13–1.30]; *p* < 0.001) and recurrence (HR = 1.49; 95%CI [1.3–1.7]; *p* < 0.001). When we superimposed the TBS on the Milan criteria, we observed that a higher TBS was associated with a higher hazard of recurrence for tumors that were exclusively within the Milan criteria (HR = 1.19; 95%CI, [1.04–1.36]; *p* = 0.013). Patients with a TBS score from 3.69 to 5.10 were variably within the Milan criteria (some within and some outside). In this population, a higher TBS was also significantly associated with a higher hazard of recurrence (HR = 1.51; 95%CI, [1.15–1.98]; *p* = 0.003; Figure 1C).

**Figure 2:**
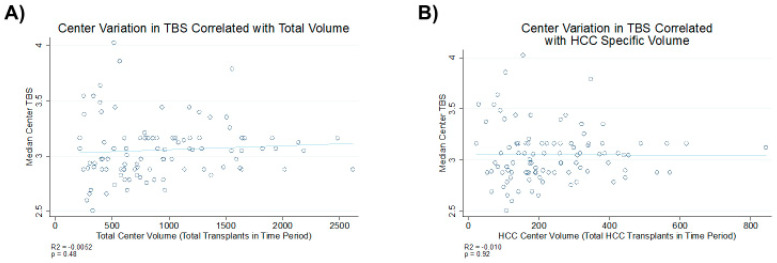
Correlation of the median TBS with (**A**) total transplant volume and (**B**) total hepatocellular carcinoma (HCC) transplant volume.

However, this trend was not observed for tumors that were exclusively outside of the Milan criteria (HR = 0.95 95%CI [0.85–1.08], *p* = 0.451). There is no correlation between the median TBS and total center case volume (total liver transplant and HCC-related liver transplants) after excluding low-volume centers (less than 13 liver transplants/year; Figure 2).

## 3. Discussion

In this study, we assessed the ability of the TBS as a solitary marker to predict oncological outcomes among patients undergoing LT for HCC. In our analysis, a high TBS was found to be a reliable predictor of worse outcomes for patients with HCC after LT. Moreover, a higher TBS was associated with higher rates of HCC recurrence in patients within or variably within the Milan criteria. The TBS did not correlate with case load across transplant centers.

Since the introduction of the Milan criteria, the field of transplant oncology has been rapidly evolving with an increasing proportion of LTs being performed for oncological indications [21,22,23,24,25,26]. However, many challenges remain, such as adequate patient selection, management of post-transplant recurrence, and refinement of neoadjuvant treatment protocols. The current literature describes multiple neoadjuvant and downstaging strategies as a bridge to LT in patients with HCC, with variable results [27,28,29,30,31,32]. Moreover, downstaged HCC patients with tumor burdens meeting United Network for Organ Sharing (UNOS) downstaging inclusion criteria had similar post-LT outcomes compared to patients transplanted within the Milan criteria [33]. Finally, a recent clinical trial showed that after effective and sustained downstaging of eligible HCCs beyond the Milan criteria, LT improved recurrence-free and overall survival of these patients compared to non-transplantation therapies [34]; thus, post-downstaging tumor response can lead to the expansion of HCC transplantation criteria.

Prognosticating outcomes in LT for HCC continues to challenge the field. Although the Milan criteria have generalized the practice of LT for HCC and have improved oncological outcomes, its predictive character has degraded with increasing candidate and oncological heterogeneity. Thus, there is an effort to transition clinical practice from dichotomous models like the Milan criteria to continuous prognostic stratification in order to improve the prediction process. A HALT–HCC score was recently proposed and was shown to have the greatest discriminatory ability for overall survival OS; C-index = 0.61 compared to all previously reported LT–HCC scores [35,36,37,38]. However, it is difficult for these scores to be implemented in daily practice as their complexity disallows easy calculation and interpretation [39]. The addition of radiographic and molecular markers in these models further increases both the accuracy and the complexity of calculating HCC-related mortality after LT [40]. A recent analysis using data from the U.S. Multicenter HCC Transplant Consortium showed that in LT patients with HCC presenting beyond the Milan criteria, successful downstaging predicted by the response of alpha-fetoprotein to locoregional therapy and tumor burden led to excellent post-LT outcomes, compared to patients without downstaging [41].

Understanding the biology and pathology of HCC is a challenge due to the cellular and anatomic complexities of the liver and the local immune environment [42], as well as due to high heterogeneity in pathogenesis, histopathology, and biological behavior [43,44]. There is an emerging body of literature that focuses on the role of tumor biology and microenvironment [30,45,46]. Thus, developing predictive models of oncological outcomes, including tumor size and focality as surrogates of tumor biology, is of paramount biological and clinical importance in patients with HCC undergoing LT. Extensive comparisons among several models predicting HCC recurrence other than the Milan criteria have been performed, showing variable results. All of them have highlighted the importance of tumor activity markers such as tumor size, tumor focality, and alpha-fetoprotein [47]. One widely used model in HCC is the Metroticket model, which captures a variety of pre-LT features of HCC that are indicative of disease biology and aggressiveness, such as alpha-fetoprotein, tumor size, and tumor number [38]. The main criticism of these models is their complexity and the fact that they cannot be easily calculated in daily practice. In this paper, we showed that the TBS, as a solitary score, can be reliable and easy to calculate. Others have shown that the TBS contributes to excellent performance of more complex scores [20,35,48] and facilitates the identification of those patients with HCC beyond the Milan criteria who will not benefit from LT [49].

The present study has several strengths and limitations. This was the largest cohort used to calculate the TBS in patients with HCC undergoing LT. Moreover, it was the first multi-institutional cohort that focused only on the TBS in a pure LT–HCC population. Additionally, we opted to analyze the TBS at the time of listing compared to the time of LT because it highlighted the value of the decision-making and intention-to-treat principles by capturing the disease burden used in the decision to transplant at the time of first referral. Of course, the study is limited due to its retrospective nature and the inherent limitation of the Scientific Registry of Transplant Recipients (SRTR) database, including the lack of explant pathology and tumor biology data. Another limitation of the study that should be mentioned is that the focus of the analysis was the oncological outcomes of LT in HCC and the prognostic value of the TBS. Of course, the overall outcomes after LT are affected by other factors such as broader eligibility criteria, including age and high-risk comorbidity profiles. In this vein, organ shortage [50] has prompted more frequent utilization of high-risk donor grafts, including those from older donors, those with hepatic steatosis, and those donated after circulatory death [51,52]. All of these factors create a complex landscape that makes it difficult to predict the success and effectiveness of certain strategies to optimize post-transplant clinical outcomes and to guide best practices [53,54].

## 4. Materials and Methods

This study used data from the SRTR. The SRTR data system includes data on all donors, wait-listed candidates, and transplant recipients in the U.S., submitted by the members of the Organ Procurement and Transplantation Network (OPTN). The Health Resources and Services Administration (HRSA), under the U.S. Department of Health and Human Services, provides oversight to OPTN and SRTR contractors. The SRTR was used to analyze all liver transplants for HCC with initial tumor size data from 2004 to 2018. The TBS was calculated based on pretransplant radiographic tumor characteristics. Tumor size was defined by the size of the largest lesion if there were multiple nodules. The TBS was defined as the distance from the origin of a Cartesian plane and comprised two variables, namely, maximum tumor size (*x*-axis) and number of tumors (*y*-axis), so that TBS^2^ = (maximum tumor diameter)^2^ + (number of tumors)^2^. For each patient, the maximum tumor diameter and the number of tumors were obtained from pretransplant imaging. The TBS was stratified for maximum sensitivity and specificity for overall and recurrence-free survival. Demographics were summarized and compared by appropriate statistical tests. Survival was compared using the Kaplan–Meier method and the logrank test, as well as the Cox proportional hazards (CPH) model (adjusted for age), the MELD score, and race. The association of the TBS with recurrence was also tested across the Milan criteria by the CPH model. To do this, we determined the TBS at which all tumors were within the Milan criteria (“all within Milan”), were variably within the Milan criteria (“variable Milan”), and were outside the Milan criteria (“all outside Milan”). We then re-ran our model on these three subsets of data and determined the HR for a one unit increase in the TBS within each subgroup. All statistical analyses were performed in STATA 15 (College Station, TX).

## 5. Conclusions

The TBS is a promising tool in predicting outcomes in patients with HCC after LT. It can capture patients within and beyond the Milan criteria who have favorable disease biology and can achieve excellent outcomes after LT. Thus, the TBS offers a reliable and simple score for estimating outcomes after LT for HCC. More complicated models offer better performance, but at the expense of being more difficult to integrate into clinical practice.

## Figures and Tables

**Table 1:** Demographics stratified by the tumor burden score.

	Low TBS (<3.1)	High TBS (≥3.1)	*p*-Value
	*n* = 6645	*n* = 5933	
Female gender, *n* (%)	1594 (24%)	1155 (19%)	<0.001
Age, median (IQR)	60.0 (55.0–64.0)	60.0 (55.0–65.0)	0.36
Race, *n* (%)			0.018
Asian	542 (8%)	536 (9%)	
Black	641 (10%)	631 (11%)	
Multi	31 (0%)	35 (1%)	
Native	36 (1%)	50 (1%)	
Pacific	23 (0%)	15 (0%)	
White	5372 (81%)	4666 (79%)	
Insurance status, *n* (%)			0.045
Public	3186 (48%)	2714 (46%)	
Private	3415 (51%)	3175 (54%)	
Self	44 (1%)	44 (1%)	
Outside of Milan criteria, *n* (%)	0 (0%)	1548 (26%)	<0.001
TBS, median (IQR)	2.4 (2.2–2.8)	3.9 (3.4–4.7)	<0.001
Positive HBV status, *n* (%)	2189 (35%)	1998 (36%)	0.19
Positive HCV status, *n* (%)	4162 (65%)	3568 (64%)	0.037
Cold Ischemia Time, median h (IQR)	6.0 (4.6–7.7)	6.0 (4.8–8.0)	0.017
Recurrence of primary malignancy, *n* (%)	460 (7%)	608 (10%)	<0.001
Death, *n* (%)	1641 (25%)	1798 (30%)	<0.001

Abbreviations: HCV, hepatitis C virus; HBV, hepatitis B virus; TBS, tumor burden score; IQR, interquartile range.

## References

[1] Beal E.W., Tumin D., Kabir A., Moris D., Zhang X.-F., Chakedis J., Washburn K., Black S., Schmidt C.M., Pawlik T.M. (2017). Trends in the Mortality of Hepatocellular Carcinoma in the United States. J. Gastrointest. Surg..

[2] Petrick J.L., Kelly S.P., Altekruse S.F., McGlynn K.A., Rosenberg P.S. (2016). Future of Hepatocellular Carcinoma Incidence in the United States Forecast Through 2030. J. Clin. Oncol..

[3] Estes C., Anstee Q.M., Arias-Loste M.T., Bantel H., Bellentani S., Caballeria J., Colombo M., Craxì A., Crespo J., Day C.P. (2018). Modeling NAFLD disease burden in China, France, Germany, Italy, Japan, Spain, United Kingdom, and United States for the period 2016–2030. J. Hepatol..

[4] Argyrou C., Moris D., Vernadakis S. (2017). Hepatocellular carcinoma development in non-alcoholic fatty liver disease and non-alcoholic steatohepatitis. Is it going to be the "Plague" of the 21st century? A literature review focusing on pathogenesis, prevention and treatment. J. BUON.

[5] Beal E.W., Tumin D., Kabir A., Moris D., Zhang X.-F., Chakedis J., Washburn K., Black S., Schmidt C.M., Pawlik T.M. (2017). Cohort Contributions to Race- and Gender-Specific Trends in the Incidence of Hepatocellular Carcinoma in the USA. World J. Surg..

[6] Forner A., Llovet J.M., Bruix J. (2012). Hepatocellular carcinoma. Lancet.

[7] Dimitroulis D., Damaskos C., Valsami S., Davakis S., Garmpis N., Spartalis E., Athanasiou A., Moris D., Sakellariou S., Kykalos S. (2017). From diagnosis to treatment of hepatocellular carcinoma: An epidemic problem for both developed and developing world. World J. Gastroenterol..

[8] Tsilimigras D.I., Bagante F., Moris D., Merath K., Paredes A.Z., Sahara K., Ratti F., Marques H.P., Soubrane O., Lam V. (2019). Defining the chance of cure after resection for hepatocellular carcinoma within and beyond the Barcelona Clinic Liver Cancer guidelines: A multi-institutional analysis of 1,010 patients. Surgery.

[9] Moris D., Felekouras E. (2017). Ignore reality but not the consequences of its ignorance: Broaden guidelines in surgery of hepatocellular carcinoma. Hepatology.

[10] Tsilimigras D.I., Mehta R., Moris D., Sahara K., Bagante F., Paredes A.Z., Farooq A., Ratti F., Marques H.P., Silva S. (2019). Utilizing Machine Learning for Pre- and Postoperative Assessment of Patients Undergoing Resection for BCLC-0, A and B Hepatocellular Carcinoma: Implications for Resection Beyond the BCLC Guidelines. Ann. Surg. Oncol..

[11] Tsilimigras D.I., Bagante F., Moris D., Ms J.M.H., Sahara K., Paredes A.Z., Mehta R., Ratti F., Marques H.P., Soubrane O. (2020). Recurrence Patterns and Outcomes after Resection of Hepatocellular Carcinoma within and beyond the Barcelona Clinic Liver Cancer Criteria. Ann. Surg. Oncol..

[12] Moris D., Vernadakis S., Papalampros A., Petrou A., Dimitroulis D., Spartalis E., Felekouras E., Fung J.J. (2016). The effect of Guidelines in surgical decision making: The paradigm of hepatocellular carcinoma. J. BUON.

[13] Gholami S., Perry L.M., Denbo J.W., Chavin K., Newell P., Ly Q., Hill C.S., Morris-Stiff G., Kessler J., Frankel T.L. (2020). Management of early hepatocellular carcinoma: Results of the Delphi consensus process of the Americas Hepato-Pancreato-Biliary Association. HPB.

[14] Moris D., Tsilimigras D.I., Kostakis I.D., Ntanasis-Stathopoulos I., Shah K.N., Felekouras E., Pawlik T.M. (2018). Anatomic versus non-anatomic resection for hepatocellular carcinoma: A systematic review and meta-analysis. Eur. J. Surg. Oncol..

[15] Mazzaferro V., Regalia E., Doci R., Andreola S., Pulvirenti A., Bozzetti F., Montalto F., Ammatuna M., Morabito A., Gennari L. (1996). Liver Transplantation for the Treatment of Small Hepatocellular Carcinomas in Patients with Cirrhosis. N. Engl. J. Med..

[16] Sasaki K., Morioka D., Conci S., Margonis G.A., Sawada Y., Ruzzenente A., Kumamoto T., Iacono C., Andreatos N., Guglielmi A. (2018). The Tumor Burden Score. Ann. Surg..

[17] Tsilimigras D.I., Moris D., Hyer J.M., Baganate F., Sahara K., Moro A., Paredes A.Z., Mehta R., Ratti F., Marques H.P. (2020). Hepatocellular carcinoma tumour burden score to stratify prognosis after resection. Br. J. Surg..

[18] Tsilimigras D.I., Mehta R., Paredes A.Z., Moris D., Sahara K., Bagante F., Ratti F., Marques H.P., Silva S., Soubrane O. (2020). Overall Tumor Burden Dictates Outcomes for Patients Undergoing Resection of Multinodular Hepatocellular Carcinoma Beyond the Milan Criteria. Ann. Surg..

[19] Tsilimigras D.I., Mehta R., Guglielmi A., Ratti F., Marques H.P., Soubrane O., Lam V., Poultsides G.A., Popescu I., Alexandrescu S. (2020). Recurrence beyond the Milan criteria after curative-intent resection of hepatocellular carcinoma: A novel tumor-burden based prediction model. J. Surg. Oncol..

[20] Sasaki K., Firl D.J., Hashimoto K., Fujiki M., Diago-Uso T., Quintini C., Eghtesad B., Fung J., Aucejo F.N., Miller C.M. (2017). Development and validation of the HALT-HCC score to predict mortality in liver transplant recipients with hepatocellular carcinoma: A retrospective cohort analysis. Lancet Gastroenterol. Hepatol..

[21] Moris D., Kostakis I.D., Machairas N., Prodromidou A., Tsilimigras D.I., Ravindra K.V., Sudan D.L., Knechtle S.J., Barbas A.S. (2019). Comparison between liver transplantation and resection for hilar cholangiocarcinoma: A systematic review and meta-analysis. PLoS ONE.

[22] Machairas N., Kostakis I.D., Tsilimigras D.I., Prodromidou A., Moris D. (2020). Liver transplantation for hilar cholangiocarcinoma: A systematic review. Transplant. Rev..

[23] Moris D., Tsilimigras D.I., Ntanasis-Stathopoulos I., Beal E.W., Felekouras E., Vernadakis S., Fung J.J., Pawlik T.M. (2017). Liver transplantation in patients with liver metastases from neuroendocrine tumors: A systematic review. Surgery.

[24] Moris D., Tsilimigras D.I., Chakedis J., Beal E., Felekouras E., Vernadakis S., Schizas D., Fung J.J., Pawlik T.M. (2017). Liver transplantation for unresectable colorectal liver metastases: A systematic review. J. Surg. Oncol..

[25] Dueland S., Syversveen T., Solheim J.M., Solberg S., Grut H., Bjørnbeth B.A., Hagness M., Line P.-D. (2020). Survival Following Liver Transplantation for Patients With Nonresectable Liver-only Colorectal Metastases. Ann. Surg..

[26] Mazzaferro V., Gorgen A., Roayaie S., Busset M.D.D., Sapisochin G. (2020). Liver resection and transplantation for intrahepatic cholangiocarcinoma. J. Hepatol..

[27] Makary M.S., Khandpur U., Cloyd J.M., Mumtaz K., Dowell J.D. (2020). Locoregional Therapy Approaches for Hepatocellular Carcinoma: Recent Advances and Management Strategies. Cancers.

[28] Gabr A., Kulik L., Mouli S., Riaz A., Ali R., Desai K., Mora R.A., Ganger D., Maddur H., Flamm S. (2020). Liver Transplantation Following Yttrium-90 Radioembolization: 15-Year Experience in 207-Patient Cohort. Hepatology.

[29] Hasan S., Abel S., Uemura T., Verma V., Koay E.J., Herman J., Thai N., Kirichenko A. (2020). Liver transplant mortality and morbidity following preoperative radiotherapy for hepatocellular carcinoma. HPB.

[30] Moris D., Rahnemai-Azar A.A., Zhang X.-F., Ntanasis-Stathopoulos I., Tsilimigras D.I., Chakedis J., Argyrou C., Fung J.J., Pawlik T.M. (2017). Program death-1 immune checkpoint and tumor microenvironment in malignant liver tumors. Surg. Oncol..

[31] Schwacha-Eipper B., Minciuna I., Banz V., Dufour J.-F.F. (2020). Immunotherapy as a downstaging therapy for liver transplantation. Hepatology.

[32] Kole C., Charalampakis N., Tsakatikas S., Vailas M.G., Moris D., Gkotsis E., Kykalos S., Karamouzis M.V., Schizas D. (2020). Immunotherapy for Hepatocellular Carcinoma: A 2021 Update. Cancers.

[33] Mehta N., Dodge J.L., Grab J.D., Yao F.Y. (2020). National Experience on Down-Staging of Hepatocellular Carcinoma Before Liver Transplant: Influence of Tumor Burden, Alpha-Fetoprotein, and Wait Time. Hepatology.

[34] Mazzaferro V., Citterio D., Bhoori S., Bongini M., Miceli R., De Carlis L., Colledan M., Salizzoni M., Romagnoli R., Antonelli B. (2020). Liver transplantation in hepatocellular carcinoma after tumour downstaging (XXL): A randomised, controlled, phase 2b/3 trial. Lancet Oncol..

[35] Firl D.J., Sasaki K., Agopian V.G., Gorgen A., Kimura S., Dumronggittigule W., McVey J.C., Iesari S., Mennini G., Vitale A. (2019). Charting the Path Forward for Risk Prediction in Liver Transplant for Hepatocellular Carcinoma: International Validation of HALTHCC Among 4,089 Patients. Hepatology.

[36] Mehta N., Heimbach J., Harnois D.M., Sapisochin G., Dodge J.L., Lee D., Burns J.M., Sanchez W., Greig P.D., Grant D.R. (2017). Validation of a Risk Estimation of Tumor Recurrence After Transplant (RETREAT) Score for Hepatocellular Carcinoma Recurrence After Liver Transplant. JAMA Oncol..

[37] Agopian V.G., Harlander-Locke M., Zarrinpar A., Kaldas F.M., Farmer D.G., Yersiz H., Finn R.S., Tong M., Hiatt J.R., Busuttil R.W. (2015). A Novel Prognostic Nomogram Accurately Predicts Hepatocellular Carcinoma Recurrence after Liver Transplantation: Analysis of 865 Consecutive Liver Transplant Recipients. J. Am. Coll. Surg..

[38] Mazzaferro V., Sposito C., Zhou J., Pinna A.D., De Carlis L., Fan J., Cescon M., Di Sandro S., Yi-Feng H., Lauterio A. (2018). Metroticket 2.0 Model for Analysis of Competing Risks of Death After Liver Transplantation for Hepatocellular Carcinoma. Gastroenterology.

[39] Moris D., Shaw B., Ong C., Connor A., Samoylova M., Kesseli S., Abraham N., Gloria J., Schmitz R., Fitch Z. (2020). A simple scoring system to estimate perioperative mortality following liver resection for primary liver malignancies—The hepatectomy risk score (HeRS). Hepatobiliary Surg. Nutr..

[40] Cucchetti A., Serenari M., Sposito C., Di Sandro S., Mosconi C., Vicentin I., Garanzini E., Mazzaferro V., De Carlis L., Golfieri R. (2020). Including mRECIST in the Metroticket 2.0 criteria improves prediction of hepatocellular carcinoma-related death after liver transplant. J. Hepatol..

[41] Kardashian A., Florman S.S., Haydel B., Ruiz R.M., Klintmalm G.B., Lee D.D., Taner C.B., Aucejo F., Tevar A.D., Humar A. (2020). Liver Transplantation Outcomes in a U.S. Multicenter Cohort of 789 Patients with Hepatocellular Carcinoma Presenting Beyond Milan Criteria. Hepatology.

[42] Moris D., Lu L., Qian S. (2017). Mechanisms of liver-induced tolerance. Curr. Opin. Organ Transplant..

[43] Refolo M.G., Messa C., Guerra V., Carr B.I., D’Alessandro R. (2020). Inflammatory Mechanisms of HCC Development. Cancers.

[44] Der Stroth L.I., Tharehalli U., Günes C., Lechel A. (2020). Telomeres and Telomerase in the Development of Liver Cancer. Cancers.

[45] Moris D., Beal E.W., Chakedis J., Burkhart R.A., Schmidt C., Dillhoff M., Zhang X., Theocharis S., Pawlik T.M. (2017). Role of exosomes in treatment of hepatocellular carcinoma. Surg. Oncol..

[46] Wang G., Wang Q., Liang N., Xue H., Yang T., Chen X., Qiu Z., Zeng C., Sun T., Yuan W. (2020). Oncogenic driver genes and tumor microenvironment determine the type of liver cancer. Cell Death Dis..

[47] Chang Y., Cho Y., Lee J.-H., Bin Lee Y., Cho E.J., Yu S.J., Sinn D.H., Kim B.H., Kim S.H., Yi N.-J. (2019). Comparison of Models for Tumor Recurrence after Liver Transplantation for the Patients with Hepatocellular Carcinoma: A Multicenter Long-Term Follow-Up Study. Cancers.

[48] Firl D.J., Kimura S., McVey J., Hashimoto K., Yeh H., Miller C.M., Markmann J.F., Sasaki K., Aucejo F.N. (2018). Reframing the approach to patients with hepatocellular carcinoma: Longitudinal assessment with hazard associated with liver transplantation for HCC (HALTHCC) improves ablate and wait strategy. Hepatology.

[49] Lai Q., Vitale A., Halazun K., Iesari S., Viveiros A., Bhangui P., Mennini G., Wong T., Uemoto S., Lin C.-C. (2020). Identification of an Upper Limit of Tumor Burden for Downstaging in Candidates with Hepatocellular Cancer Waiting for Liver Transplantation: A West–East Collaborative Effort. Cancers.

[50] Dutkowski P., Linecker M., DeOliveira M.L., Müllhaupt B., Clavien P.-A. (2015). Challenges to Liver Transplantation and Strategies to Improve Outcomes. Gastroenterology.

[51] Gao Q., Mulvihill M.S., Scheuermann U., Davis R.P., Yerxa J., Yerokun B.A., Hartwig M.G., Sudan D.L., Knechtle S.J., Barbas A.S. (2019). Improvement in Liver Transplant Outcomes From Older Donors. Ann. Surg..

[52] Taylor R., Allen E., Richards J.A., Goh M.A., Neuberger J., Collett D., Pettigrew G.J., Goh A.M., Liver Advisory Group to NHS Blood and Transplant (2019). Survival advantage for patients accepting the offer of a circulatory death liver transplant. J. Hepatol..

[53] Moris D., Shaw B.I., Gloria J., Kesseli S.J., Samoylova M.L., Schmitz R., Manook M., McElroy L.M., Patel Y., Berg C.L. (2020). Textbook Outcomes in Liver Transplantation. World J. Surg..

[54] Muller X., Marcon F., Sapisochin G., Marquez M., Dondero F., Rayar M., Doyle M.M.B., Callans L., Li J., Nowak G. (2018). Defining Benchmarks in Liver Transplantation. Ann. Surg..

